# ADVANCING DATA COLLECTION AND SHARING FOR PERSON-CENTERED DEMENTIA CARE IN LOW-RESOURCE LONG-TERM CARE SETTINGS

**DOI:** 10.1093/geroni/igae098.0569

**Published:** 2024-12-31

**Authors:** Alison Rataj, Eleanor McConnell

**Affiliations:** Chair; Discussant

## Abstract

Person-centered care (PCC) is essential to support people living with dementia and is a guiding principle in care services. Inequities in PCC and dementia care access persist in long-term care (LTC) settings, including poorer urban and rural communities, which have fewer healthcare resources. While growing evidence has demonstrated the consequences of LTC inequities, research is limited in understanding strategies to improve dementia care in low-resource settings. This symposium features four presentations from the Advancing Person-Centered Dementia Care in Low-Resource Contexts Project emphasizing unique challenges and opportunities for providing PCC in low-resource LTC settings from the perspectives of administrators, direct care staff, residents with dementia and their families. The first presentation reports results from a community-based, participatory research study in four LTC settings in federally designated medically underserved areas highlighting what matters when providing quality dementia care from the perspectives of LTC leaders, staff, residents and their families. The second presentation shares PCC strategies used by direct care workers stratified by length of tenure in the workforce. The third presentation examines factors related to measuring PCC quality with a specific focus on measurement in low-resource settings. The fourth presentation summarizes results from a series of design thinking workshops with family members, staff, and leaders in low-resource LTC settings to co-create processes for supporting high-quality PCC. This symposium reveals innovative strategies to provide dementia care and generates future directions for addressing inequities in low-resource LTC settings. Common Data Elements for International Research in Residential Long-term Care Interest Group Sponsored Symposium

